# Dissociable sources of erogeneity in social touch: Imagining and perceiving C-Tactile optimal touch in erogenous zones

**DOI:** 10.1371/journal.pone.0203039

**Published:** 2018-08-24

**Authors:** Elena Panagiotopoulou, Maria Laura Filippetti, Antje Gentsch, Aikaterini Fotopoulou

**Affiliations:** 1 University College London, Research Department of Clinical, Educational & Health Psychology, London, United Kingdom; 2 Centre for Brain Science, Department of Psychology, University of Essex, Colchester, United Kingdom; 3 Department of Psychology, Ludwig-Maximillians University, Munich, Germany; University of Chicago, UNITED STATES

## Abstract

Previous research points to two major hypotheses regarding the mechanisms by which touch can be experienced as erotogenic. The first concerns the body part to which touch is applied (erogenous zones) and the second the modality of touch (sensual touch optimal in activating C Tactile afferents). In this study, we explored for the first time the relation between those two mechanisms in actual and imagined social touch. In a first experiment, we randomly assigned “Giver” and “Receiver” roles within 19 romantic couples (20 females, 18 males, age 32.34 ± 8.71SD years) and asked the “Giver” to apply CT-optimal (3 cm/s) vs. CT-suboptimal (18 cm/s) touch on an erogenous (neck) vs. non-erogenous zone (forehead) of their partner. We then obtained ratings of pleasantness and sexual arousal from both “Receivers” and “Givers”. In a second experiment, 32 healthy females (age 25.16 ± 5.91SD years) were asked to imagine CT-optimal vs. CT-suboptimal stimulation (stroking vs. patting) and velocity (3 cm/s vs. 18 cm/s) on different erogenous vs. non-erogenous zones and rate pleasantness. While both erogenous body part and CT-optimal, sensual touch were found to increase pleasant and erotic sensations, the results showed a lack of an interaction. Furthermore, pleasantness was induced by mere imagination of touch without any tactile stimulation, and touch that was sexually arousing for the receiver was rated as more sexually arousing for the giver as well, pointing to top-down, learned expectations of sensory pleasure and erogeneity. Taken together, these studies provide the first direct evidence that while both the body location to which touch is applied and the mode of touch contribute to pleasant and erotic sensations, these two factors appear to mediate subjective pleasantness and erogeneity by, at least partly, independent mechanisms.

## Introduction

Social touch is a fundamental part of intimate human relationships, signalling and communicating a wide range of distinct emotions [1,2]. Skin-to-skin touch in adults can also be erotogenic, that is, it can generate feelings of sexual arousal. However, there is disagreement regarding the neurophysiological and psychological mechanisms by which the erogeneity of touch is experienced and communicated. Specifically, there are currently two major hypotheses regarding such mechanisms, the first emphasising the (erogenous) body part to which touch is applied and the second stressing the (affective) modality of touch that is used. To our knowledge however, no study has addressed the relation between these two hypotheses. We first outline each of these hypotheses below, and then we present how this study aimed to address their relation.

The concept of bodily ‘erogenous zones’ refers to certain body parts, such as the genitalia, that are relevant to sexual behaviours and hence tactile stimulation of these body parts leads to heightened, erotic responses. Yet, various body parts that are not near the genitalia can elicit strong erotic sensations when being touched. To explain this paradox, it was initially proposed that erotic sensations from these body parts arise as a consequence of their adjacency to the genitals in the somatotopic map of the primary somatosensory cortex (S1) [3]. Despite this intriguing possibility, a recent systematic investigation of erogenous zones, involving 41 different body areas, provided evidence against this hypothesis [4]. Specifically, there were no significant inter-correlations between ratings of sexual arousal for nearby S1 sites, suggesting that sexual arousal is unrelated to proximity of cortical representation of body parts in S1. Moreover, there were surprisingly low sexual arousal ratings for the feet, which are adjacent to the cortical mapping for genitals in S1. Therefore, it was suggested that the somatotopy of erogenous zones may be coded elsewhere in the brain, possibly in the insular cortex [4], as this area is involved in the re-representation of interoception offering the basis for subjective feelings [5,6]. Nevertheless, there has been no direct exploration of this hypothesis as yet.

Interestingly, the insula is also implicated in a second, alternative hypothesis about the mechanisms of tactile erogeneity. This hypothesis links erogeneity to the functional role of a specific tactile modality. In fact, there is evidence for a specialised system coding for dynamic touch, involving slow-conducting, unmyelinated peripheral nerve fibers, the so-called CT (C Tactile) afferents that have been differentiated from the fast-conducting, myelinated peripheral nerve fibers (Ab fibers) typically studied in discriminatory touch [7–9]. This type of touch is referred to as “affective” or “sensual” touch. Studies using single unit microneurography, a neurophysiological method employed to record impulse conduction directly from peripheral nerve fibers of the skin, have shown that CT-afferents respond optimally to dynamic touch applied at very low indentation forces ranging from 0.3 to 2.5 mN [10]. Further, there is a distinct spatial pattern due to the fact that CT fibers are found only in hairy skin, while they are lacking in glabrous skin, suggesting that this type of touch is perceived differently across body regions. Moreover, they are temperature specific—showing a preference for a temperature of approximately 32°C, which corresponds to interpersonal skin-to-skin contact [11]. Crucially, the CT-afferents respond optimally to a specific range of stimulation velocities (1–10 cm/s) and less strongly at slower or faster velocities leading to an inverted U shape response pattern [12]. Stimulating the skin at CT-optimal velocities is also accompanied by stronger feelings of subjective pleasure as compared to stimulating the skin in suboptimal velocities [12]. This correlation between CT sensitivity and subjective ratings of pleasantness suggests that CT-afferents represent a distinct peripheral ascending pathway for pleasant tactile stimulation [12]. However, CT-optimal touch is not merely associated with feelings of pleasantness, but it has also been found to be associated with feelings of erotic sensation [13–15]. Specifically, the same inverted U-shaped relationship found between stroking velocities and pleasantness ratings has also been found between stroking velocities and eroticism ratings, with CT-optimal velocities (1-10cm/s) leading to higher levels of perceived eroticism than slower or faster velocities [14–15].

Furthermore, research suggests that the discriminative and affective properties of touch are also processed differently in the brain. Functional magnetic resonance imaging (fMRI) analysis during CT stimulation has shown activation not only in the classic somatosensory areas S1 and S2 (as with discriminative touch) but also in the posterior insular cortex [7–9]. Furthermore, when applying soft stroking to neuropathy subjects lacking Ab afferents, only the posterior insular cortex is activated [7, 16]. Neural responses in other brain regions have also been correlated with soft stroking, such as the superior temporal sulcus, the orbitofrontal cortex, the medial prefrontal cortex and the anterior cingulate cortex [17–19]. Moreover, repetitive transcranial magnetic stimulation (rTMS) studies have shown that inhibition of right hemisphere S1 and S2 does not affect pleasantness perception associated with affective touch [20–21]. Thus, these studies confirm the idea that affective touch is associated with activation of a multimodal neural network of brain areas mapping body state representation and emotion, including the insula, that bypasses the somatosensory areas S1 and S2 (but see [22]).

Despite the fact that both proposals regarding the origins of erogeneity, namely somatotopy and CT-afferents, stress the role of the insular cortex in the perception of erotic touch, they differ as regards to whether the body part or the modality of touch are the critical factors in the erogeneity of touch. Yet to our knowledge, no previous study has assessed the relationship between these two hypotheses when either receiving or, giving touch. The primary aim of this study was to assess this question by manipulating in the same design both the erogeneity of body parts and the modality of touch used to elicit pleasant and erotic sensations.

Moreover, a secondary aim of this study was to assess the relationship between body part and modality of touch in eliciting pleasant sensations when tactile stimulation was merely imagined, rather than actually perceived. In an intimate context, tactile interactions may be influenced not only by current sensations, but also by expectations, imagination and memories. Indeed, it has been shown that erotic mental imagination is strong enough to cause sexual arousal [23]. Interestingly, there is evidence that the anterior insula responds both when CT-optimal touch is experienced and imagined, without any sort of tactile or even visual stimulation [24]. However, no study has tested whether imagining CT-optimal (i.e. sensual) touch in erogenous versus non-erogenous zones can also give rise to subjective feelings of pleasantness. Accordingly, a secondary aim of the present study was to assess the relationship between body part and modality of touch in eliciting pleasant sensations when tactile stimulation was merely imagined.

In sum, previous research points to two major hypotheses regarding the mechanisms by which touch can be experienced as erotogenic. The first concerns the body part to which touch is applied (erogenous zones) and the second the modality of touch (CT-optimal, sensual touch). In this study, we explored for the first time the relation between these two hypotheses both when touch was actually given and received but also when it was merely imagined. In a first experiment, we investigated actual (active and passive) stimulation of an erogenous vs. non-erogenous zone with CT-optimal versus non-optimal touch and measured both pleasantness and erotic perception of touch in romantic couples. We hypothesised that CT-optimal, sensual touch would be rated as more pleasant and more arousing than neutral touch, and that sensual touch on an erogenous zone would be more pleasant and arousing that sensual touch on a non-erogenous zone. In a second experiment, we investigated pleasantness perception of imagined sensual vs. neutral touch in erogenous vs. non-erogenous zones. We hypothesised that CT-optimal, sensual touch would be perceived as more pleasant than neutral touch, and that pleasantness would be higher when it is imagined being applied on an erogenous zone as compared to a non-erogenous zone.

## Experiment 1: Pleasant and erotic perception of active and passive sensual touch on erogenous zones

### Method

#### Ethical declarations

The study was approved by the Ethics Committee of the Research Department of Clinical, Educational and Health Psychology, University College London.

#### Participants

Twenty-three romantic couples gave their informed consent to participate in the study. Participants were visitors of a public event on haptics at the Royal Institution in London. Three couples were excluded as they failed to follow the experimental instructions during administration and one couple was excluded due to an experimental error during administration. The final sample comprised of 19 couples, 20 females and 18 males, for a total of N = 38 participants (age 32.34 ± 8.71SD years). All subjects gave written, informed consent.

#### Design

The experiment employed a 2x2x2 design with 2 within-subject factors: 1) Body Part (neck vs. forehead), 2) Velocity (3 cm/s, i.e. CT-optimal vs. 18 cm/s, i.e. CT-suboptimal) and one between-subject factor, Role (Giver vs. Receiver).

#### Apparatus and material

The experimental paradigm (the velocity factor, in particular) was explicitly developed based on the large body of evidence revealed by microneurography (see Introduction) on CT-fiber properties underlying interpersonal touch [12]. Before the experimental task, the experimenter assigned touch “Giver” and “Receiver” roles within the couple, following a standard randomisation procedure. These roles where then kept constant throughout the experiment. The experimenter trained the Giver to apply the different types of touch (CT-optimal and CT-suboptimal) using the index and middle fingers of the participant’s dominant hand, with a velocity of either 3cm/s (slow/CT-optimal) or 18 cm/s (fast/CT-suboptimal). To help the Giver better understand the difference between slow and fast stroke velocities, the experimenter applied both touches on the Giver’s forearm during the training phase. The body parts to which touch was applied were selected from a list of 41 body parts generated by a recent systematic review [4]. This list was divided into 3 almost-equal parts and we subsequently selected one body part from the top 14 (i.e. Nape of Neck) and one body part from the bottom 14 (i.e. Forehead) to represent erogenous and non-erogenous zones, respectively. The highest-rated body parts were the ones that are strongly associated with sexual content (i.e. genitals, breasts) and were not selected for ethical and practical considerations. Both participants were given a booklet, which contained the pleasantness and sexual arousal ratings, to be completed after each trial.

#### Procedure

Prior to the main experimental phase, participants were familiarized with procedures and all rating scales. The Receiver was asked to sit on a chair facing an empty wall to avoid any external distraction. The experimenter then identified the stroking areas on the nape of the neck and forehead of the Receiver, each measuring 9 cm long × 4 cm wide, and marked them with a washable marker on their skin. In each trial, the Giver was asked to deliver a “stroking-type” touch of one of the two trained velocities, either on the neck or forehead of their partner, as instructed by a checklist that the experimenter held. After each trial, both the Giver and the Receiver were asked to silently rate both the pleasantness and sexual arousal value of the touch in a continuous VAS scale (-10 to 10; *not at all* to *extremely*) using the booklet provided to each so that they could not see, or hear each other’s ratings. That is, Givers were asked to rate how pleasant and sexually arousing it was to apply the touch, whereas Receivers were asked to rate how pleasant and sexually arousing was to feel the touch. Before starting the 12 trials task, the couple underwent 2 practice trials.

#### Data analysis

As data were not normal, we computed z-scores for both the pleasantness and sexual arousal ratings. We performed two separate analyses of variance (ANOVA) for each dependent variable (Pleasantness and Arousal ratings). In both analyses, the Body Part (“Neck” vs. “Forehead”) and Velocity (“Slow” vs. “Fast”) were entered as within-subject variables, whereas Role (“Giver” vs. “Receiver”) and Gender were the between-subject factors. Greenhouse–Geisser correction was applied when sphericity could not be assumed (Mauchly’s test for sphericity, p = 0.05). Comparisons were assessed for significance using planned two-tailed t-tests. Level of significance was set to 0.05. Information on the relationship between pleasantness and sexual arousal is included in Supplementary Material (S1 File).

### Results

#### Pleasantness ratings

**Fig 1:**
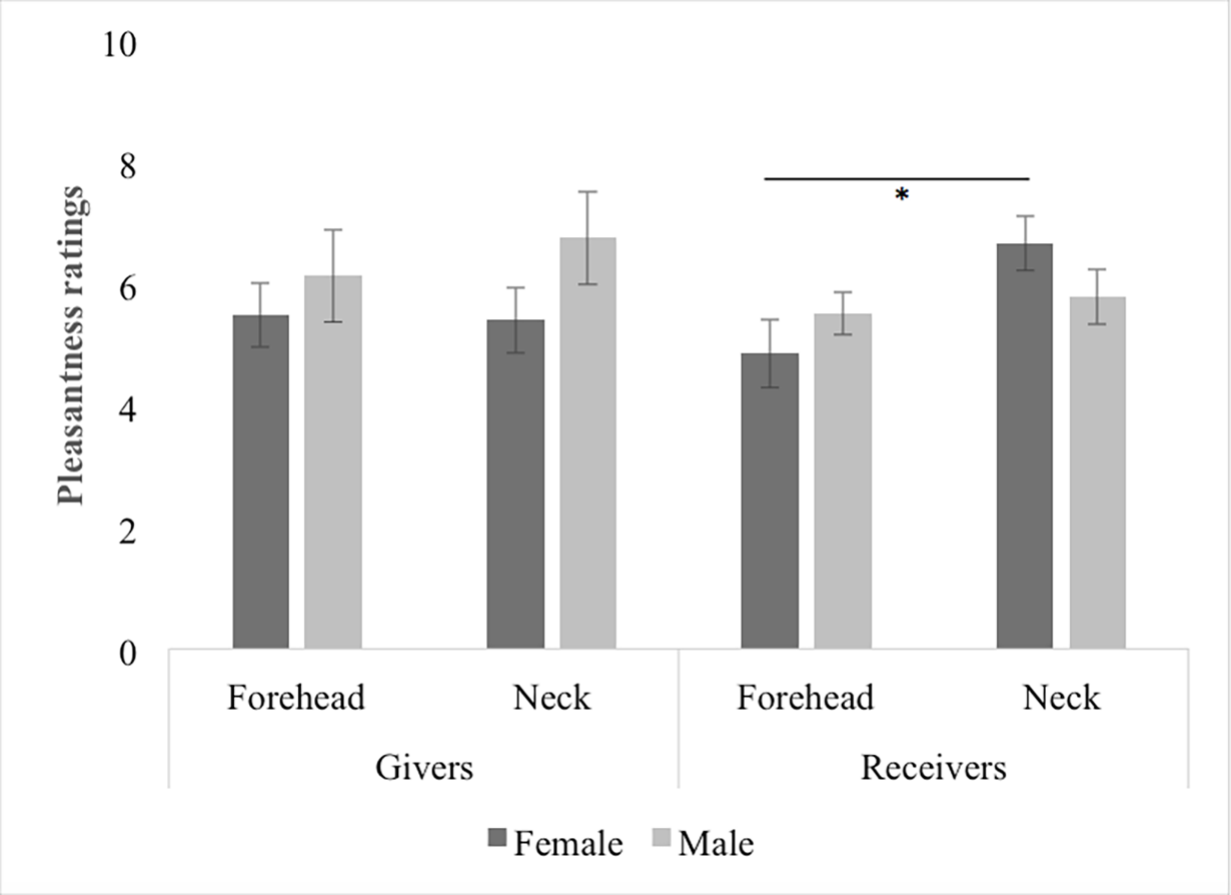
Pleasantness ratings of givers and receivers. The graph shows that, among Givers, there was no significant difference between males and females in pleasantness ratings on the neck or the forehead. Among Receivers, females preferred more to be touched on the neck compared to the forehead. Error bars indicate standard error.

The ANOVA revealed significant main effects of Velocity, F(1,34) = 109.07, *p* < 0.001 and Body Part, F(1,34) = 15.10, *p* < 0.001, with CT optimal touch being rated as more pleasant than CT-suboptimal touch, t(37) = 10.44, *p* < 0.001 and touch on the neck being judged as more pleasant compared to touch on the forehead, t(37) = 3.05, *p* = 0.004. All other interactions were not significant (S1 File). The interaction Body Part x Role was significant, F[1,34] = 5.20, *p* = 0.029, with Receivers rating the touch on the neck as more pleasant than the touch on the forehead, t(18) = 3.05, *p* = 0.007, but no difference between these body parts was found in Givers, t(18) = 1.09, *p* = 0.288. We also found a significant interaction Body Part x Role x Gender, F[1,34] = 11.17, *p* = 0.002. To better understand this three-way interaction, we averaged ratings across Velocity and investigated the judged pleasantness among Givers and Receivers separately. We found that, among Givers, there was no significant difference between males and females in pleasantness ratings on the neck or the forehead, Body Part x Gender: F [1,17] = 0.087, *p* = 0.771. However, among Receivers we found a significant interaction between Body Part and Gender, F[1,17] = 7.61, *p* = 0.013, suggesting that females preferred significantly more to be touched on the neck compared to the forehead, t(8) = -3.77, *p* = 0.006 (Fig 1).

#### Sexual arousal ratings

**Fig 2:**
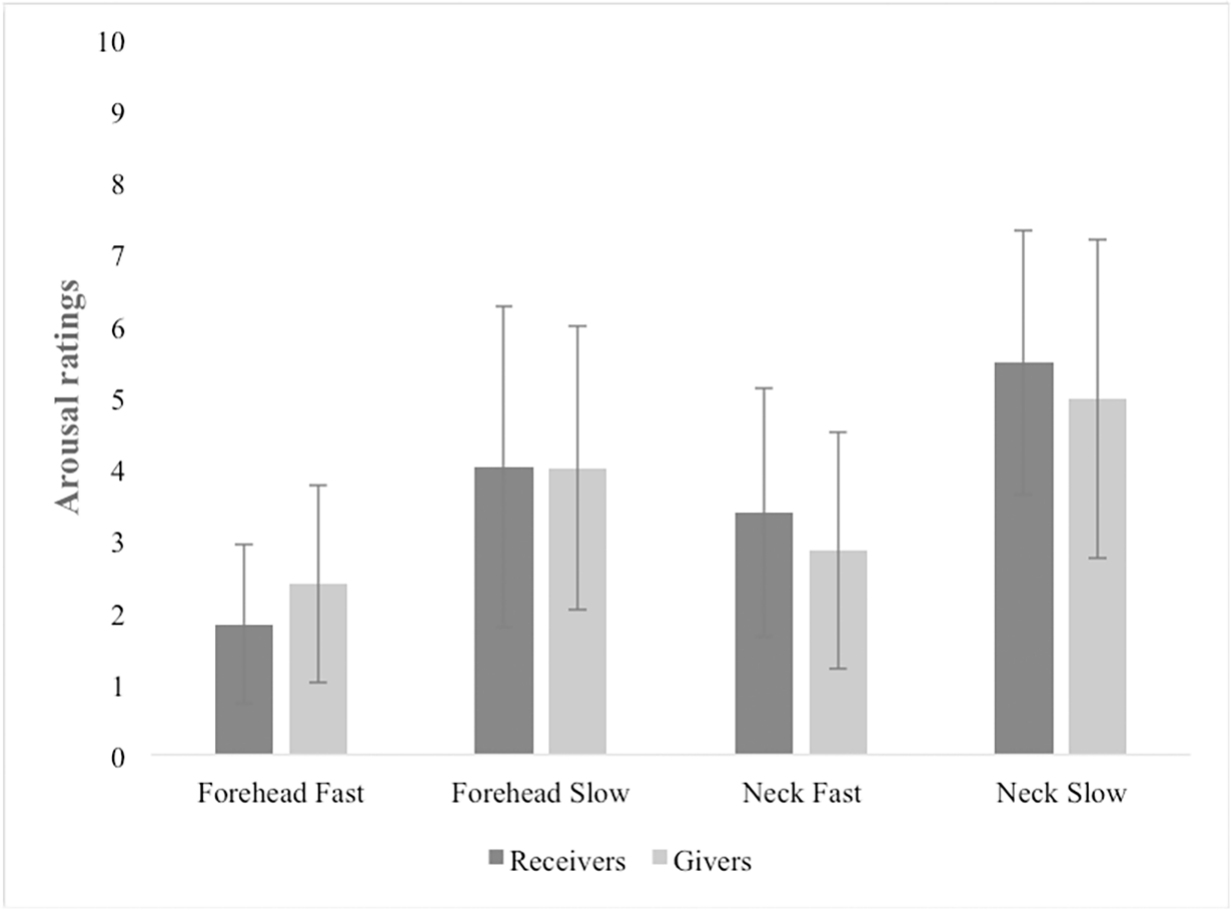
Arousal ratings of givers and receivers for the neck and forehead. The graph shows that there was no preference in arousal ratings between Receivers and Givers. Error bars indicate standard error.

Gender did not reveal any significant effects, and therefore data were averaged across this factor. The ANOVA revealed significant main effects of Velocity, F[1,36] = 107.60, *p* < 0.001 and Body Part, F[1,36] = 4.14, *p* < 0.001. Couples rated as more arousing to feel and give touch on the neck, compared to the forehead, t(37) = 5.81, *p* < 0.001, and to feel and give the CT optimal touch, compared to the CT non-optimal touch, t(37) = 10.42, *p* < 0.001. We also found an interaction trend between Body Part and Role, F[1,36)] = 4.14, *p* = 0.049. Planned independent samples t-tests however showed that no significant difference in arousal ratings between neck and forehead was present among the two groups (Givers, t(36) = 0.581, *p* = 0.565; Receivers, t(36) = -1.040, *p* = 0.306) (Fig 2). All the other interactions were not significant (S1 File).

## Experiment 2: Pleasant perception of imagined sensual touch on erogenous zones

### Method

#### Ethical declarations

The study was approved by the Ethics Committee of the Research Department of Clinical, Educational and Health Psychology, University College London.

#### Participants

Thirty-two right-handed healthy females (age 25.16 ± 5.91SD years) were recruited from UCL Psychology Subject Pool and took part in the study for course credit or £8/hour. Even though this experiment did not involve actual touch, a female experimenter was instructing participants to imagine different types of touch. Research has shown that the hedonic value of touch varies according to the gender of both receiver and giver [22], therefore, to control for gender effects related to touch perception and for practical considerations, only heterosexual females took part in the study. Participants had no known physical or mental illness and normal sense of touch. All subjects gave written, informed consent.

#### Design

We employed a 2x2x2 within-subjects design with 3 factors: 1) Type of Stimulation (stroking, i.e. CT-optimal vs. patting, i.e. CT-suboptimal, 2) Velocity (3 cm/s, i.e. CT-optimal vs. 18 cm/s, i.e. CT-suboptimal) and 3) Body Part (erogenous vs. non-erogenous zone). The dependent measure was reported pleasantness of imagined touch on a scale from -100 (not at all) to +100 (extremely).

The experiment also included two manipulation checks. First, at the beginning and end of each trial we asked participants to report their subjective pleasantness and sexual arousal in response to video clips showing different types of touch. Here, we explored the perceptual effects of seen touch irrespective of body part, and manipulated “Type of Stimulation” and “Velocity”. Second, we explored whether the sample perceived the chosen body parts as highly or minimally erogenous. Hence, we manipulated “Body part” (erogenous vs. non-erogenous) and measured the reported sexual arousal.

#### Apparatus and material

The experimental paradigm was explicitly developed based on the large body of evidence on CT-fiber properties underlying interpersonal touch [12]. As with experiment 1, the body parts were selected from the same list of 41 body parts [4]. Three body parts were selected from the top 14 and three body parts from the bottom 14 to represent erogenous and non-erogenous zones, respectively. Therefore, we selected the erogenous zones “Nape of neck”, “Lower back” and “Stomach”, whereas the non-erogenous zones were “Forearm”, “Forehead” and “Elbow”.

To familiarise participants with the different types of touch, we presented four videos for the four types of touch (slow stroking, fast stroking, slow patting, fast patting) (S1 Fig). In all familiarisation videos, the stimulated area was a human forearm (6 cm length and 4 cm width), with light pressure (targeting at indentation forces of < 0.3 N). Each video clip lasted 4 sec. The number of pats was matched to the number of strokes for both the slow and fast condition, with 2 touches in the “slow touch” videos and 12 touches in the “fast touch” videos. The four conditions (slow stroking, fast stroking, slow patting, fast patting) were repeated three times each, for a total of 12 trials, the order of which was randomised.

#### Procedure

Computer-generated stimulation was controlled by a customized software program (Presentation software, Neurobehavioral Systems Inc.) and presented on the screen, which was placed at a viewing distance of approximately 80 cm. Each of the 12 trials began with one of the familiarisation videos in order to demonstrate the type of touch to imagine. After seeing the video, participants were asked to rate the pleasantness of the touch they watched (“How pleasant is this type of touch?”) in a continuous VAS scale (-100 to 100; *not at all* to *extremely*). Right after rating each video, the main experimental task took place, whereby participants were asked to imagine the same type of touch they saw in the video being applied to various body parts (“Imagine how pleasant the touch would be on each of the following six body parts”) and rate pleasantness in a continuous VAS scale (-100 to 100; *not at all* to *extremely*).

After the main experimental task, participants were presented again with each of the four familiarisation videos showing the different types of touch and were asked to rate the sexual arousal of the touch irrespective of body part (“How sexually arousing is this type of touch?”), in a continuous VAS scale (-100 to 100; *not at all* to *extremely*). Then, they were asked to rate the sexual arousal value of each of the six body parts irrespective of type of touch (“How sexually arousing is this body part?”), once again in a continuous VAS scale (-100 to 100; *not at all* to *extremely*).

#### Data analysis

Statistical analyses were performed using SPSS v. 23 (IBM, Chicago, IL, USA). For the main experimental task (imagined touch), pleasantness ratings were averaged across the 3 trials of each condition. A repeated-measures ANOVA was conducted with Type of Stimulation, Velocity and Erogeneity of Body Part as within-subject factors. Pleasantness was measured and post hoc analyses were performed using Bonferroni correction. Level of significance was set to 0.05. Information on how data of our two manipulation checks were analysed is included in Supplementary Material (S1 File).

### Results

**Fig 3:**
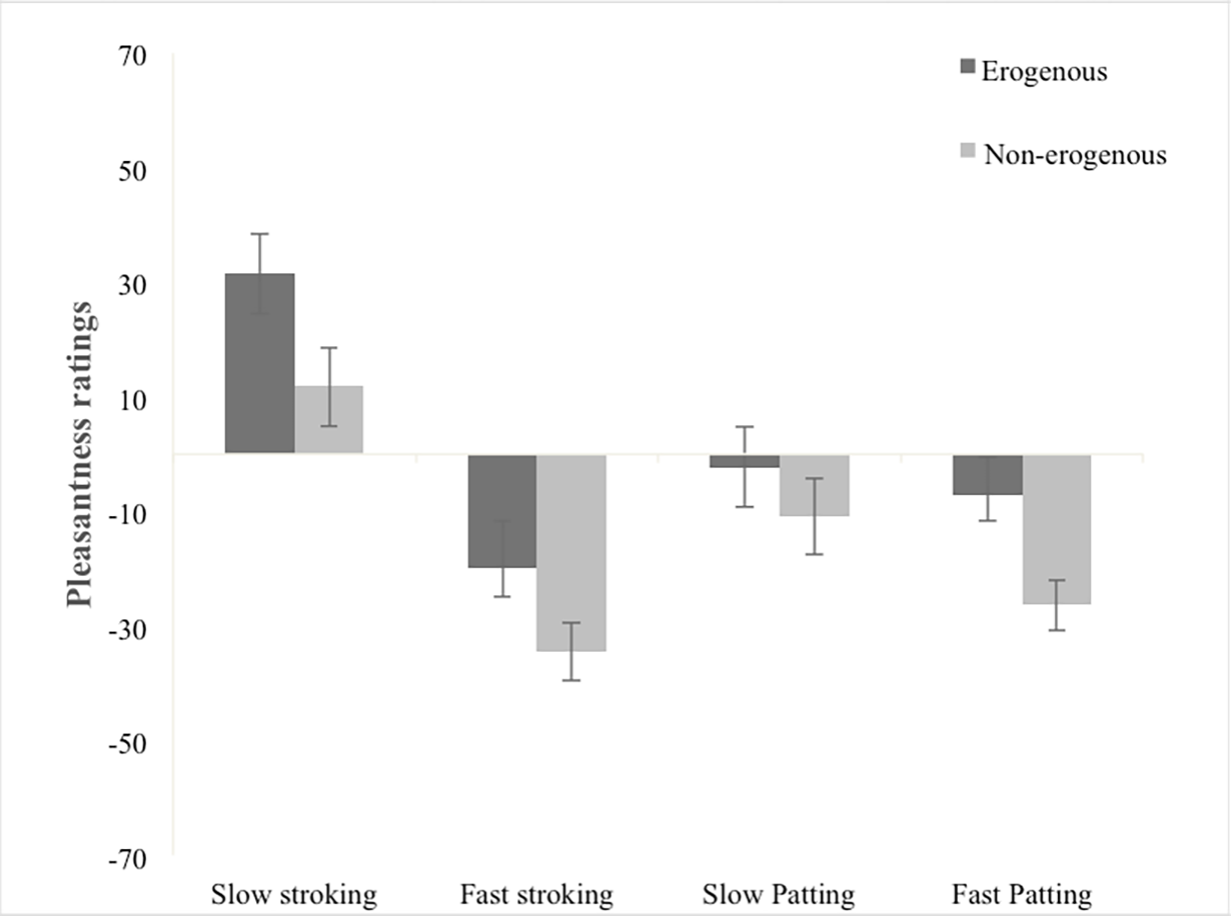
Pleasantness ratings for different types of imagined touch in erogenous vs. non-erogenous zones. The graph shows that slow stroking was perceived as more pleasant than all other types of touch and that for slow stroking and fast patting pleasantness was higher for erogenous as compared to non-erogenous zones. Error bars indicate standard error.

Based on the findings of the second manipulation check (details in S1 File), “Stomach” and “Forearm” were excluded from this analysis. Therefore, pleasantness ratings for imagined touch were averaged for the 2 high-rated and the 2 low-rated erogenous zones. The ANOVA revealed significant main effects of Type of Stimulation, F[1,31] = 4.85, *p* = 0.035, as well as Velocity, F[1,31] = 28.68, *p* < 0.001, and Body Part, F[1,31] = 8.00, *p* = 0.008. The three-way interaction between Type of Stimulation, Velocity and Body Part was significant, F[1,31] = 4.87, *p* = 0.035, as well as the two-way interaction between Type of Stimulation and Velocity, F[1,31] = 28.74, *p* < 0.001. None of the other two-way interactions were significant (Type of Stimulation and Body Part, F[1,31] = 0.639, *p* = 0.430; Velocity and Body Part, F[1,31] = 0.543, *p* = 0.467). Post hoc analyses revealed that for both high and low erogenous zones, pleasantness ratings for slow stroking were significantly higher than fast stroking, slow patting and fast patting (all p-values < 0.001). For slow stroking and fast patting, pleasantness ratings for erogenous zones were significantly higher than for non-erogenous zones, [slow stroking: t(31) = 2.87, *p* = 0.007]; [fast patting: t(31) = 3.17, *p* = 0.003] (Fig 3).

### Discussion

Interpersonal sensual touch between individuals can convey different meanings, depending on how it is performed and in which body part it is applied. At present, the neurophysiological and psychological mechanisms by which the erogeneity of touch is experienced and communicated are still a matter of debate. In fact, it is unclear whether it is the (erogenous) body part to which touch is applied [4] or the (affective) modality of touch that is used that drives our pleasant and erotic perception [14–15]. This study explored the relationship between body part and tactile modality in eliciting pleasant and erotic sensations, by using the socially-relevant CT-afferent system tuned to affective touch, in order to examine the perceptual effects of imagined and actual sensual vs. neutral touch in erogenous vs. non-erogenous zones.

In Experiment 1 we explored actual stimulation of an erogenous (neck) vs. non-erogenous zone (forehead), with the aim of assessing subjective pleasantness and erogeneity. We tested romantic couples, which allowed us to address the reciprocal property of touch in an ecologically valid setup. In line with previous research, the results showed that sensual touch was perceived as more pleasant and more sexually arousing than neutral touch. Similarly, we also found that touch on the neck (erogenous) was rated as more pleasant and sexually arousing compared to touch on the forehead (non-erogenous). However, the lack of interaction suggests that CT-optimal stroking (i.e. modality of touch) and touch on erogenous zones (i.e. body part) may mediate subjective pleasantness and erogeneity by at least partly independent mechanisms.

In Experiment 2, we explored pleasantness perception of imagined sensual (CT-optimal) vs. neutral (CT-suboptimal) stimulation of erogenous vs. non-erogenous zones. Our findings provide the first direct evidence that imagining sensual touch was perceived as more pleasant than all other types of touch, suggesting that pleasantness induced by merely imagining touch may be driven, at least in part, by top-down processes, guided by learned expectations of sensory pleasure [25–26]. In fact, interpersonal touch is generally present in romantic interactions, therefore most people have memories of the pleasant state that sensual, CT-optimal stimulation elicits. In contrast to previous findings [14, 27], we also found that pleasantness was higher when participants were asked to imagine sensual stimulation being applied on erogenous as compared to non-erogenous zones. The lack of an interaction in this second experiment also suggests that CT-optimal stroking (i.e. modality of touch) and touch on erogenous zones (i.e. body part) may mediate subjective pleasantness of imagined touch by at least partly independent mechanisms.

In addition to the findings regarding our main hypotheses, in Experiment 1 we found no differences between touch-receivers and givers in how they reported their subjective sexual arousal ratings for CT-optimal vs. CT-suboptimal touch and for neck vs. forehead. In our sample, arousal ratings had higher variance compared to pleasantness ratings, which could explain the absence of significant differences in our analyses. Variability in arousal across subjects is not surprising, given the known impact of contextual and individual factors on erotic perception [28–29]. Interestingly however, and indeed despite this individual variability, our additional correlation analyses (S1 File) show that both across body parts and stroking velocity, participants that rated the touch as highly arousing also found the same touch as pleasant. Additionally, across participants we found positive associations in arousal ratings of givers and receivers in both erogenous and non-erogenous zones, as well as for CT-optimal stroking velocity. Previous research has reported hedonic benefits of CT-optimal touch for the giver [25]. More specifically, there is evidence that stroking others’ skin feels softer and smoother than stroking one’s own skin. This so-called “social softness illusion” is selectively present when touch is applied according to the optimal properties of the receiver’s CT-based affective touch system, i.e. optimal velocities and hairy skin only [25]. Here we suggest that similar mechanisms might be in place in the context of CT-mediated erotic touch. Activating the neurophysiological system for sensual touch in the receiver, appeared to induce feelings of sexual arousal to the giver as well, reflecting a mechanism of sensual sharing between individuals.

In addition, gender differences were found in the way males and females reported the experience of receiving touch in different body parts. Women rated touch on the neck as more pleasant than similar touch on the forehead. This finding is in line with the recent evidence that while participants of both genders equally rated the forehead as not sexually arousing, the mean score of level of arousal for the neck was higher for females [4], pointing to potentially higher levels of sensitivity to the neck in women. However, considering our relatively small sample size, future research should replicate and provide support to these findings.

Our data support the presence of an interplay between affective touch and erogenous zones, possibly mediated by other factors, such as gender differences and context. Neuropsychological and behavioural studies have now widely demonstrated the effects of affective tactile interactions for affiliative behaviour, social bonding, and emotion regulation [1, 30–32]. Yet touch can have different impacts on others, depending on contextual factors and situational variables. Research has shown that CT touch might scaffold rather than determine erotic perception, by creating a sort of emotional backdrop to sexual feelings [14]. Therefore, other factors should be taken into account when considering erotic touch perception and this might be especially true in the context of a romantic relationship. For example, tactile interactions in a newly-established romantic relationship may be perceived as more erotic compared to the same touch in longer-term couples. In fact, previous studies have found that sexual arousal ratings are negatively associated with relationship length [33] and that people in a long-term relationship are less sexually active than newly established couples [14].

Neuroimaging studies have shown that CT-optimal touch specifically activates the insula [7], and that a map in this brain region could potentially offer an explanation for the arrangement of the erogenous zones [4]. The insula has been recently identified as the cortical hub for the primary interoceptive information about the physiological condition of the body [34, 35]. Evidence also points to the insula in integrating interoceptive states with other exteroceptive, cognitive and social information, providing the basis for all subjective feelings from the body and even emotional awareness [5, 34]. While pleasant and erotic perception of sensual touch in erogenous zones could be associated with activity in anterior insula, as mentioned above, the absence of an interaction between touch modality and body part in both experiments (i.e. with actual and imagined touch) suggests that different cognitive mechanisms might be responsible for the conscious experience of pleasantness and erogeneity. More specifically, it seems that CT-optimal touch on non-erogenous zones, and vice versa, is still perceived to be pleasant and erotic, suggesting that pleasantness and erogeneity of touch can be elicited by both mechanisms, together or in isolation. Future studies could establish whether these two sources of erogeneity are influenced differentially by contextual factors, such as gender or relationship nature/quality. Moreover, the involvement of neural circuits was not directly tested, hence a conclusion about the neural pathways for conscious perception of touch sensuality cannot be reached. Further neuroimaging research is, therefore, required to address this.

Altogether, this study provides the first direct evidence that while *how* we touch and *where* we touch are both crucial in eliciting pleasant and erotic sensations, they represent dissociable sources of pleasantness and erogeneity in social touch. Furthermore, sensory pleasure and erogeneity seem to be driven at least partly by top-down, learned expectations. In fact, pleasantness is induced by mere imagination of touch without any tactile stimulation. Nevertheless, for this experiment only women were tested to control for gender effects related to touch [22], thus, future studies could investigate whether the present results also extend to women instructed by male experimenter and men instructed by female and male experimenters. Further evidence for the role of top-down factors in erotic perception is provided by the fact that there seems to be a strong sensual reciprocity of giving and receiving touch among romantic couples. This intersubjective communication and the subsequent erotogenic benefits for both receiver and giver may therefore act as a reinforcer for interpersonal touch and affiliative behaviours. Future studies could use autonomic measures, such as skin conductance and heart rate, to explore the mechanisms of interpersonal synchronisation between touch-givers and receivers, which has been found to be crucial for social behaviours [36] and tease apart other top-down factors (e.g. expectations) that could influence pleasant and erotic perception of touch.

## Supporting information

S1 Data Data file for Experiment 1 and 2. (XLSX)

S1 Fig Still image from the familiarisation video. (TIF)

S1 File Supplementary material. (DOCX)
