# Supplementary figures and images for: Dissociable sources of erogeneity in social touch: Imagining and perceiving C-Tactile optimal touch in erogenous zones
Source: PLoS One. 2018 Aug 24;13(8):e0203039. doi: 10.1371/journal.pone.0203039 (PMC6108496; doi:10.1371/journal.pone.0203039)

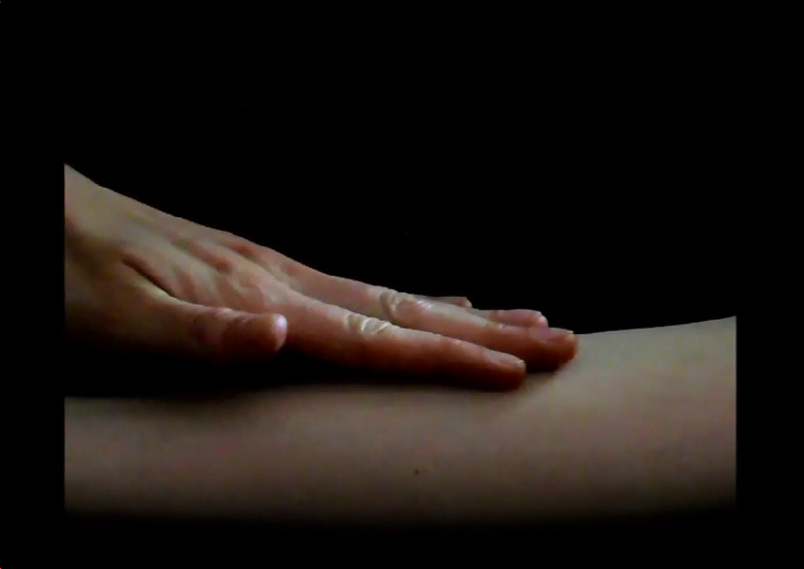

Supplement: S1 Fig — (TIF) [file pone.0203039.s002.tif]
