# Supplementary material for: Dissociable sources of erogeneity in social touch: Imagining and perceiving C-Tactile optimal touch in erogenous zones
Source: PLoS One. 2018 Aug 24;13(8):e0203039. doi: 10.1371/journal.pone.0203039 (PMC6108496; doi:10.1371/journal.pone.0203039)
Supplement: S1 File — (DOCX) [file pone.0203039.s003.docx]

**Experiment 1** – **Non significant effects (ANOVA)**

1. Dependent variable: Pleasantness ratings

| Interaction | F | p-value |
| --- | --- | --- |
| Velocity x Role | 2.334 | 0.136 |
| Velocity x Gender | 2.042 | 0.162 |
| Velocity x Role x Gender | 0.013 | 0.909 |
| Area x Gender | 0.316 | 0.222 |
| Velocity x Area | 0.022 | 0.671 |
| Velocity x Area x Role | 0.081 | 0.414 |
| Velocity x Area x Gender | 0.002 | 0.902 |
| Velocity x Area x Role x Gender | 0.043 | 0.552 |

Table 1. Non-significant interactions of the ANOVA for the pleasantness ratings

1. Dependent variable: Arousal ratings

| Interaction | F | p-value |
| --- | --- | --- |
| Velocity x Role | 0.609 | 0.440 |
| Velocity x Body Part | 0.395 | 0.534 |
| Velocity x Body Part x Role | 0.888 | 0.352 |

Table 2. Non-significant interactions of the ANOVA for the arousal ratings

**Experiment 1** - **Correlation between pleasantness and sexual arousal**

In order to better understand the relationship between pleasantness and sexual arousal in erogenous vs non-erogenous zones under CT-optimal vs CT-suboptimal touch, we ran a series of correlation analyses between the ratings of the two body parts (neck and forehead) separately and between CT-optimal and suboptimal touch separately. First, we looked at possible associations in pleasantness and arousal ratings in the two different body parts. With regard to the erogenous zone (i.e. neck), we found that ratings of pleasantness were positively correlated with ratings of sexual arousal, r = 0.625, *p* < 0.001. Similarly, pleasantness ratings on the forehead were positively correlated with sexual arousal ratings on the same non-erogenous zone, r = 0.609, *p* < 0.001. Hence, these results suggest that pleasantness and arousal ratings seem to go hand-in-hand. That is, the higher the pleasantness ratings on a body part, the higher the perceived sexual arousal on the same body part.

Next, we investigated whether couples perceived CT-optimal and suboptimal touch similarly pleasant and arousing. Indeed, for both the CT-optimal and suboptimal touch, we found that the higher the pleasantness ratings, the higher the reported sexual arousal; CT-optimal touch, r = 0.548, *p* < 0.001; CT-suboptimal touch, r = 0.492, *p* < 0.001. In line with a recent study (Bendas et al 2017), these results suggest that erotic and pleasant touch are similarly perceived at velocities that are known to optimally vs. suboptimally activate CT fibers.

Finally, we investigated whether givers’ pleasantness and arousal ratings in erogenous vs non-erogenous zones, as well as for CT-optimal vs CT-suboptimal touch was associated to receivers’ ratings. We found that, while arousal ratings between givers and receivers were positively correlated in both erogenous (neck) (r = 0.557, *p* < 0.001) and non-erogenous (forehead) conditions (r = 0.363, *p* = 0.025), only in the case of forehead touch there was a positive association between givers’ and receivers’ pleasantness ratings, r = 0.504, *p* = 0.001. The equivalent correlation in neck touch was not significant (r = 0.218, *p* = 0.189). Interestingly, with regard to the CT-optimal stroking velocity, we found that the higher the reported arousal by the giver, the higher the experienced sexual arousal for the receiver, r = 0.403, *p* = 0.012. The equivalent correlation in CT-suboptimal stroking velocities was not significant (r = 0.906, *p* = 0.568).

**Experiment 2 - Manipulation checks**

Data analysis

For the first manipulation check (seen touch), a repeated-measures analysis of variance (ANOVA) was conducted with Type of Stimulation and Velocity as within-subject factors. Pleasantness and sexual arousal were measured. Post hoc analyses were performed using Bonferroni correction. For the second manipulation check (body parts), the data for sexual arousal ratings of body parts were not normal, therefore a non-parametric Kruskal-Wallis H test was conducted, followed by a Mann-Whitney test. Level of significance was set to 0.05.

Results

*Manipulation check 1: Pleasantness and sexual arousal ratings for seen touch (irrespective of body part)*

**Pleasantness ratings** after watching the different types of touch in the videos revealed significant main effects of Type of Stimulation, F[1,31] = 15.51, *p* < 0.001 and Velocity, F[1,31] = 48.33, *p* < 0.001. Additionally, we found a significant interaction between Type of Stimulation and Velocity, F[1,31] = 50.38, *p* < 0.001. Post hoc analyses revealed that pleasantness ratings were significantly higher for slow stroking as compared to fast stroking, slow patting and fast patting (all p-values < 0.001) (Figure 1). Similarly, **sexual arousal ratings** of observed touch revealed significant main effects of Type of Stimulation, F[1,31] = 34.39, *p* < 0.001, and Velocity, F[1,31] = 56.66, *p* < 0.001. The analysis also showed a significant interaction between Type of Stimulation and Velocity, F[1,31] = 56.57, *p* < 0.001. Post hoc analyses revealed that sexual arousal ratings were significantly higher for slow stroking as compared to fast stroking, slow patting and fast patting (all p-values < 0.001) (Figure 2).

*Manipulation check 2: Sexual arousal ratings for different body parts*

For sexual arousal ratings of different body parts, there was an overall significant difference between body parts: χ^2^(5) = 86.70, *p* < 0.001. The means of the erogenous zones were significantly higher than the means of the non-erogenous zones: U = 1411.50, *p* < 0.001. Reported sexual arousal was significantly different from zero in the expected direction for all body parts, except for “Stomach”**,** t(31) = 1.96, *p* = 0.059 and “Forearm”**,** t(31) = -0.960, *p* = 0.345 (Figure 3).

**Figure 1** | Pleasantness ratings for different types of seen touch. The graph shows that slow stroking was perceived as more pleasant than the other types of touch. Error bars indicate standard errors.

**Figure 2** | Sexual arousal ratings for different types of seen touch. The graph shows that slow stroking was perceived as more sexually arousing than the other types of touch. Error bars indicate standard errors.

**Figure 3** | Sexual arousal ratings for different body parts. The graph shows that erogenous zones were perceived as more sexually arousing than non-erogenous zones. Error bars indicate standard errors.
